# Virtual NBI image synthesis using stable diffusion for enhanced recognition of early gastric cancer: a technical validation study

**DOI:** 10.1080/07853890.2025.2523565

**Published:** 2025-06-28

**Authors:** Changda Lei, Xiuji Kan, Yifan Ouyang, Yutong Mei, Yunbo Guo, Kaicheng Hong, Junbo Li, Bilin Wang, Rui Li

**Affiliations:** ^a^ Department of Gastroenterology, The First Affiliated Hospital of Soochow University, Suzhou, China; ^b^ Suzhou Institute of Biomedical Engineering and Technology, Chinese Academy of Science, Suzhou, China

**Keywords:** Artificial intelligence, deep learning, early gastric cancer, narrow band imaging, stable diffusion

## Abstract

**Background:**

Narrow band imaging (NBI) can assist endoscopists in detecting early gastric cancer (EGC) more easily, but its widespread use is hindered by economic cost and technical property rights. We aim to realize the conversion of white light endoscopy (WLE) images into virtual narrow band imaging (Vir–NBI) images using stable diffusion.

**Methods:**

Endoscopic images were retrospectively collected from 325 patients who underwent endoscopic submucosal dissection (ESD). A total of 273 NBI images from 218 patients were used to fine-tune stable diffusion, which then converted 111 WLE images from 107 patients into Vir–NBI images. Endoscopists assessed the images and evaluated their effectiveness in diagnosing EGC and depicting lesion margins in the form of WLE, NBI, and Vir–NBI image pairs.

**Results:**

Compared with WLE images, Vir-NBI images have better quality. The accuracy of junior endoscopists in diagnosing EGC by observing WLE images alone, simultaneous WLE and NBI images, and simultaneous WLE and Vir–NBI images were 61.26%, 79.28% and 81.08%, respectively. For intermediate endoscopists, the diagnostic accuracy was 72.07%, 86.79% and 84.68%, respectively. For senior endoscopists, the diagnostic accuracy was 80.18%, 95.50% and 92.79%, respectively. In addition,Vir-NBI images had higher area concordance rate andsuccessful whole-lesion diagnosis than WLE images (43.85% vs 39.32%, *p* < 0.001) (45.33% vs 32.87%, *p* < 0.001).

**Conclusions:**

Vir–NBI images has similar observation effect with real NBI image, which helps endoscopists better visualize the lesion structure, thus improving the accuracy of EGC diagnosis.

## 1. Introduction

According to the study, about 20 million new cases of malignant tumors were diagnosed globally in 2022, of which gastric cancer (GC) was the fifth most common malignant tumor in terms of both incidence and death rate [1]. Early gastric cancer (EGC) is defined as tumor tissue confined to the mucosa and submucosa, with or without metastases [2]. The overall prognosis of GC patients is poor, with 5-year survival rates of only 14.0–45.6% for patients with pathologic stages III–IV, whereas EGC patients not only have a 5-year survival rate of more than 90% after endoscopic treatment [3,4]. This shows the importance of timely detection of EGC.

Currently, white light endoscopy (WLE) is the most commonly used diagnostic procedure for EGC [5]. However, some EGC lesions are difficult for endoscopists to detect in WLE images due to objective reasons such as small extent, flat morphology, and shallow depth of invasion of some EGC lesions [6–8]. Related studies have shown that GC has a misdiagnosis rate ranging from 2.0% to 40.0% in WLE examination [9–12]. This indicates that the diagnostic effect of WLE on EGC is not satisfactory. In recent years, image enhanced endoscopic technology has made rapid progress. Narrow-band imaging (NBI), a representative optical enhancement technique, utilizes specialized bandpass filters to exclusively transmit 415 nm (blue) and 540 nm (green) wavelengths. These selected spectral bands correspond to hemoglobin’s absorption maxima while demonstrating limited tissue penetration depth, thereby providing high-contrast visualization of superficial mucosal microvasculature and fine morphological features [13]. The vessel plus surface (VS) classification system is a method to judge whether malignant lesions occur by evaluating the changes of mucosal microvessels and microstructure. NBI can help endoscopists diagnose through VS classification system by virtue of its advantages. Compared to WLE, NBI has been proven to have superior performance in identifying EGC [14–17]. However, due to reasons such as technological property rights and economic costs, endoscopists in many underdeveloped areas or small medical institutions are unable to use NBI to observe and detect lesions. Therefore, there is a current need to invent a technology that can convert WLE images into NBI images to help endoscopists better observe lesions and reduce the risk of missed diagnosis of EGC.

CycleGAN is an artificial intelligence (AI) technology that can perform image transformation [18], it has played an important role in the application of digestive diseases. Yuan et al. used CycleGAN to convert WLE images of colorectal polyps in dynamic videos into Virtual indigo carmine (VIC) images for observation [19]. A research team from Japan used CycleGAN to convert WLE images into VIC images for 3D reconstruction of the stomach [20]. The VIC images generated by Suzuki et al. using CycleGAN can help determine the subtypes of gastric tumors [21]. Although CycleGAN performs well in the field of image translation, the quality of the generated images is often limited, the details are not detailed enough, and may be accompanied by artifacts and pattern collapse issues [22].

In contrast, stable diffusion, as a generative model based on stepwise denoising reversion, can more accurately capture data distributions and generate images with high levels of detail and authenticity. Additionally, stable diffusion’s robust training process mitigates the instability commonly seen in adversarial models, particularly in complex medical image tasks, offering significant advantages. Currently, the stable diffusion model has been widely applied in various medical imaging tasks, including cross-modal image translation, image reconstruction, image generation, denoising, and anomaly detection [23].

To the best of our knowledge, there is currently no research on using stable diffusion for endoscopic image conversion. In this study, we utilized stable diffusion, a model with strong conversion capabilities, to generate virtual-narrow band imaging (Vir–NBI) images from WLE images. The aim is to enable more endoscopists, especially those without hardware support, to use virtual NBI images for detailed observation and timely detection of EGC during endoscopic examinations. Finally, we compared the efficacy of Vir–NBI images with WLE and NBI images in identifying lesion structures and diagnosing EGC.

## 2. Methods

### 2.1. Patients and materials

We retrospectively collected medical records and endoscopic images of 325 patients with gastric mucosal lesions who underwent endoscopic submucosal dissection (ESD) treatment at the First Affiliated Hospital of Soochow University from January 2016 to June 2024. According to the revised Vienna Classification of Gastrointestinal Epithelial Diseases [24], the pathological types of lesions are diagnosed as EGC in categories 3 (high-grade intraepithelial neoplasia), 4 (intramucosal carcinoma), and 5 (submucosal carcinoma), while categories 1 (negative for neoplasia) and 2 (low-grade intraepithelial neoplasia) are diagnosed as noncancerous lesions. All WLE and NBI images were captured by standard endoscopes (GIF-Q260J, GIF-HQ290, GIF-H290Z Olympus Corporation, Tokyo, Japan) and EVIS LUCERA ELITE endoscopic system (CV-290, Olympus Corporation).

This study adopts a retrospective design, and the use of research data has been submitted in writing to the Science and Technology Department of the First Affiliated Hospital of Soochow University, and has been approved by the Ethics Committee after review (Ethical Approval Number:2024-649).

### 2.2. Dataset preprocessing and experimental design

**Figure 1.**
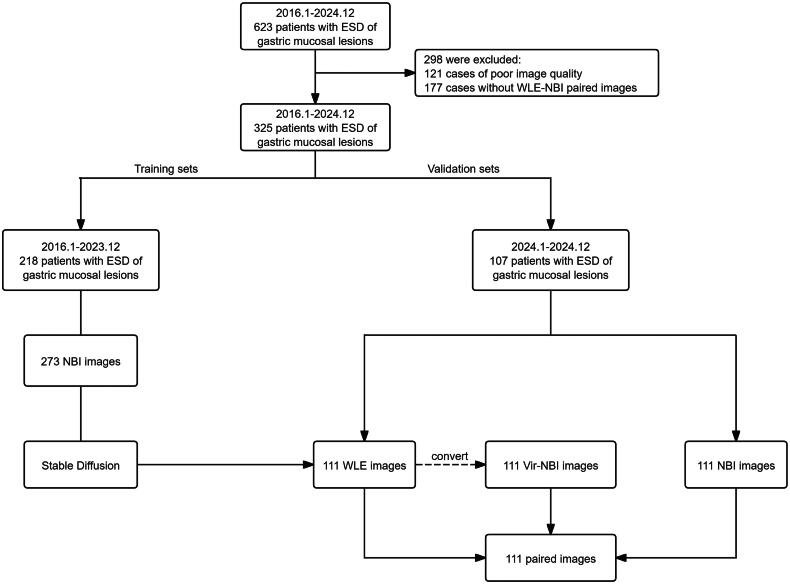
Flowchart of training and validation dataset.

A doctoral student excluded patients with poor image quality (bleeding, halo, blur, and defocusing) and those who did not simultaneously retain WLE and NBI images of the lesion site. The filtered WLE and NBI images were saved in BMP or JPEG format, uniformly cropped, with excess black background removed, retaining only the images within the endoscopic field of view, and then saved in PNG format. 273 NBI images from 218 patients were selected to fine-tune stable diffusion, and Vir–NBI images were generated from the WLE images of another 107 patients to validate the effectiveness of stable diffusion in the form of WLE, NBI, and Vir NBI image pairs (Figure 1).

### 2.3. Generate Vir–NBI images through stable diffusion conversion

Stable diffusion, as a deep learning based generative model, is capable of generating high-quality images based on text prompts or other conditional inputs. In this study, we utilize stable diffusion to convert WLE images into NBI images in gastrointestinal endoscopy.

**Figure 2.**
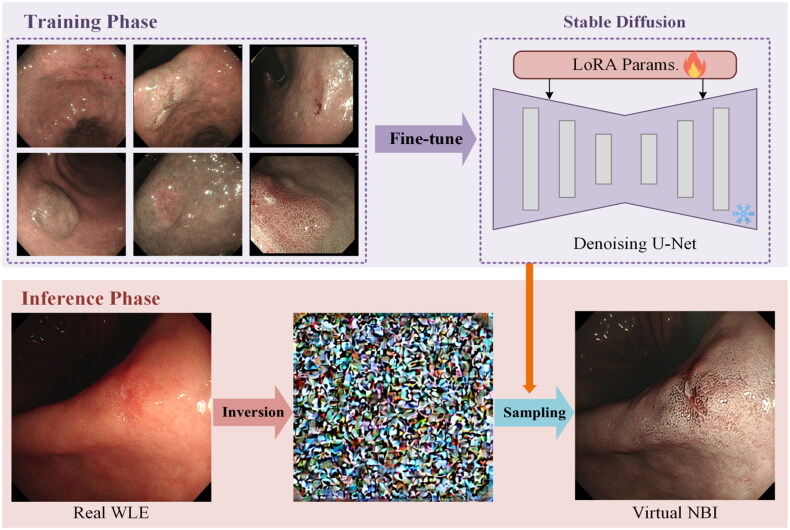
Stable diffusion converts WLE images into Vir–NBI images.

Our virtual NBI synthesis framework is built upon the stable diffusion model and consists of two main stages: LoRA-based fine-tuning and self-attention injection sampling. The framework is illustrated in Figure 2. In the fine-tuning stage, we adapt the U-Net backbone of the stable diffusion model using a small curated dataset of real NBI images, guided by the text prompt *‘narrow-band imaging, NBI’*. The model is optimized by minimizing the mean squared error between the predicted noise and the actual Gaussian noise, enabling it to capture the visual appearance and modality-specific semantic patterns of NBI images.

In the subsequent sampling stage, we propose a self-attention injection mechanism to guide the generative process. Specifically, during DDIM-based sampling, the self-attention maps of the LoRA fine-tuned model are dynamically replaced with those from the original pre-trained stable diffusion model applied to the corresponding WLE image. This process starts at the first sampling timestep and is maintained throughout generation, allowing the model to preserve the semantic structure of the WLE input while accurately mapping it to the NBI modality.

All experiments were conducted using Python 3.8 and PyTorch 1.11.0, with acceleration provided by CUDA 11.3 on an NVIDIA RTX 4090D GPU with 24 GB VRAM. LoRA was applied to fine-tune the attention layers of the U-Net with a rank of 16. The model was trained for 100 epochs using the AdamW optimizer with a learning rate of 2 × 10^−4^ and weight decay of 0.01. During inference, DDIM inversion with *T*_1_ = 1000 steps is first used to recover the latent representation of the WLE image, followed by *T*_2_ = 50 guided sampling steps using the same text condition as in fine-tuning. On average, the generation of a single Vir–NBI image takes total 27.8 s, with 20.6 s for DDIM inversions and 7.2 s for sampling costs (Figure 2).

### 2.4. Compared with CycleGAN

To quantitatively validate the superiority of the proposed stable diffusion-based method over traditional GAN-based approaches, we conducted a comparative study using CycleGAN as a representative baseline. We evaluated the quality of generated virtual NBI images using four metrics: Fréchet Inception Distance (FID), Learned Perceptual Image Patch Similarity (LPIPS), Artifact-aware FID (ArtFID), and Structural Similarity Index Measure (SSIM).

The results demonstrate that the stable diffusion model consistently outperforms CycleGAN across all metrics. For instance, our method achieved a lower FID (12.56 vs 13.47), indicating higher fidelity to real NBI distributions. Similarly, LPIPS and ArtFID scores were significantly reduced (LPIPS: 0.32 vs 0.39; ArtFID: 17.88 vs 20.09), reflecting better perceptual similarity and fewer visual artifacts. Additionally, our method obtained a higher SSIM (0.26 vs 0.22), further confirming its ability to preserve structural details.

These findings support that the stable diffusion framework effectively alleviates common problems associated with GANs, such as mode collapse and artifact introduction, leading to the generation of more realistic and semantically consistent virtual NBI images.

### 2.5. Outcome measurements

#### 2.5.1. Observation quality of surface structure, surface blood vessels, and lesion margins in WLE, NBI, and Vir–NBI images

Blind evaluation of WLE, NBI, and Vir–NBI images in the validation set was conducted by two experienced endoscopists with over 10000 cases of endoscopic examination experience. The evaluation focused on lesion surface structure, surface blood vessels, and lesion edges, with rating divided into three levels: poor, moderate, and good. For surface glandular ducts and surface blood vessels, ‘poor’ refers to difficult to identify morphology; ‘moderate’ refers to a form that is easy to observe but difficult to recognize, while ‘good’ refers to a form that is easy to recognize. For lesion margins, poor means the lesion is difficult to distinguish from the surrounding normal tissue, moderate means the lesion can be identified as an area and the edge can be inferred, and good means the edge between the lesion and the normal tissue is clear and distinguishable. In case of disagreement, the two endoscopists will discuss further until a consensus is reached.

#### 2.5.2. The accuracy of WLE, NBI, and Vir–NBI image diagnosis for EGC

Detailed observations of WLE, NBI, and virtual NBI images in the validation set were conducted by three junior endoscopists (with less than 5 years of endoscopic examination experience), three intermediate endoscopists (with 5–10 years of endoscopic examination experience), and three senior endoscopists (with more than 10 years of endoscopic examination experience), respectively. Endoscopists within the same seniority independently observed WLE images, WLI+NBI images, and WLI+Vir–NBI images, and finally determined whether the lesion was malignant (EGC) through consensus negotiation. The diagnostic accuracy for EGC of different observation methods was then compared across endoscopists with varying years of experience.

#### 2.5.3. The area concordance rate and successful whole-lesion diagnosis

**Figure 3.**
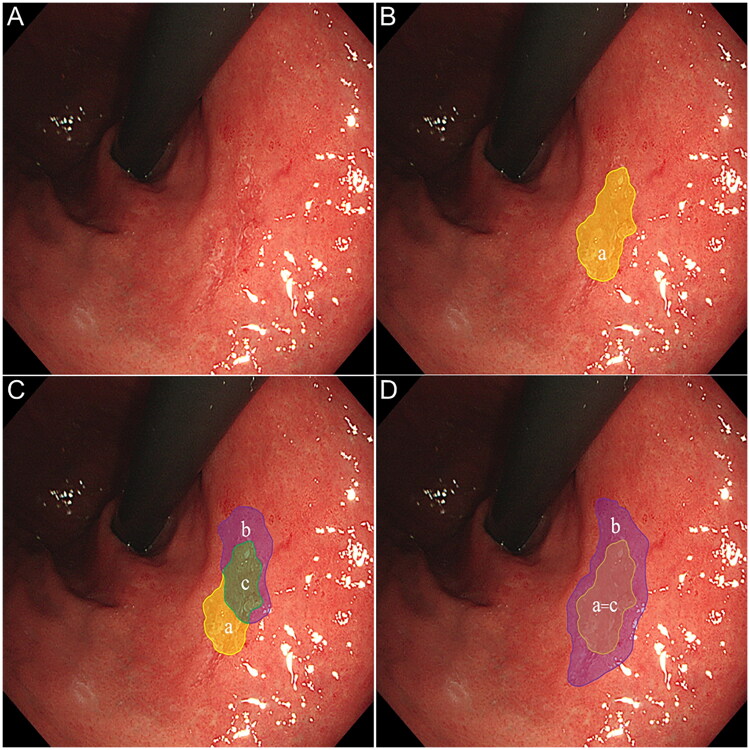
Definition of the area concordance rate detection and successful whole-lesion diagnosis. (A) Original endoscopic image before annotation. (B) Region a (yellow) is the pathological gold standard range. (C) Region b (purple) represents the lesion area delineated by the endoscopist; region c (green) represents the intersection of the pathological gold standard and the scope defined by the endoscopist. (D) The endoscopist’s delineation of the lesion area b (purple) completely covers the pathological gold standard area a (yellow).

Use Labelme software to randomly display all 111 paired images on a computer monitor, including 111 WLE images, 111 NBI images, and 111 Vir–NBI images. Three junior endoscopists, three intermediate endoscopists, and three senior endoscopists delineated the lesion boundaries on each WLE and Vir–NBI image in the validation set. Then, compare the lesion areas designated by endoscopists of different seniority with the pathological gold standard after ESD. The area concordance rate is defined as the ratio of the overlapping area between the lesion range defined by the endoscopist and the lesion range under the pathological gold standard to the collection area. The calculation formula is *c*/(*a* + *b*–*c*)×100%. The definition of successful whole-lesion diagnosis is defined as the lesion scope delineated by the endoscopists fully covering the lesion range under the pathological gold standard (Figure 3).

### 2.6. Statistical analysis

The quality evaluation of WLE, NBI, and Vir–NBI images was compared using Friedman test and Steel–Dwass test. The McNemar test is used to compare the accuracy of diagnosing EGC by observing WLE images, WLE + NBI images, and WLE+Vir–NBI images. Mann–Whitney *U* test is used to analyze the area concordance rate. Pearson chi-square test is used to analyze the successful diagnosis rate of the entire lesion. *p* < 0.05 is considered statistically significant. All analyses were conducted using JMP version 17.0 (SAS Institute, Cary, NC, USA).

## 3. Results

### 3.1. Patient characteristics

In the training sets, the average age of patients was 63.00 ± 8.96 years. There were 148 males (67.89%) and 70 females (32.11%). In the validation sets, the average age of patients was 65.83 ± 9.41 years. There were 72 males (67.29%) and 35 females (32.71%). Detailed information on the location, pathology, invasion depth, differentiation type, etc. of the lesion is shown in Table 1.

**Table 1.** Baseline characteristics of patients.

**Characteristics**	**Training sets (*n* = 218)**	**Validation sets (*n* = 107)**
**Age, mean ± SD, years**	63.00 ± 8.96	65.83 ± 9.41
**Sex, *n* (%)**		
Male	148 (67.89%)	72 (67.29%)
Female	70 (32.11%)	35 (32.71%)
**Specimen size, mean ± SD, cm**	3.87 ± 1.34	3.77 ± 1.36
**Morphology^b^, *n* (%)**		
Elevated type (0–I, 0–IIa)	56 (25.69%)	31 (28.97%)
Flat type (0–IIb)	29 (13.30%)	16 (14.95%)
Depressed type (0–IIc, 0–III)	65 (29.82%)	36 (33.65%)
Mixed type (0-IIa + IIc, etc)	68 (31.19%)	24 (22.43%)
**Location, *n* (%)**		
Upper 1/3	32 (14.68%)	25 (23.36%)
Middle 1/3	96 (44.03%)	45 (42.06%)
Lower 1/3	90 (41.29%)	37 (34.58%)
**Pathology, *n* (%)**		
Inflammation	3 (1.38%)	2 (1.87%)
Intestinal metaplasia	2 (0.92%)	0 (0.00%)
Low-grade intraepithelial neoplasia	40 (18.35%)	22 (20.56%)
High-grade intraepithelial neoplasia	54 (24.77%)	25 (23.36%)
Adenocarcinoma	119 (54.59%)	38 (35.51%)
**Invasion depth of EGC, *n* (%)**		
Intraepithelial neoplasia^a^		
Intramucosal carcinoma	151 (87.70%)	73 (87.95%)
Submucosal carcinoma	22 (13.30%)	10 (12.05%)
**Histologic type of EGC^a^*n* (%)**		
Differentiated type	150 (87.28%)	73 (87.95%)
Undifferentiated type	23 (12.72%)	10 (12.05%)

EGC early gastric cancer, SD standard deviation.

^a^According to the Paris endoscopic classification.

^b^According to the Nakamura classification.

### 3.2. Comparison of WLE, NBI, Vir–NBI image quality evaluation

**Figure 4.**
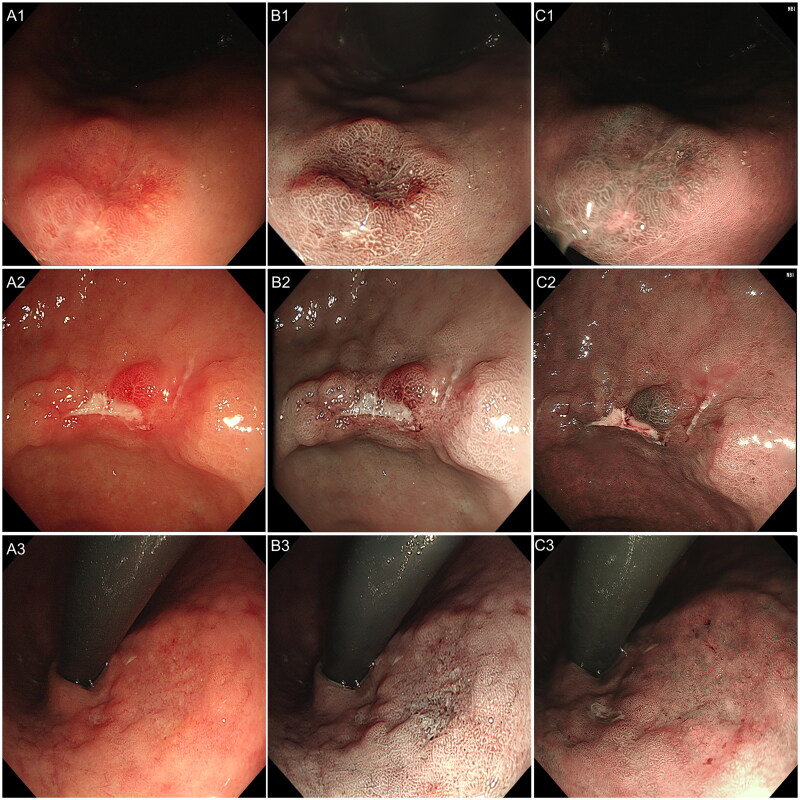
Schematic diagram of the same lesion in WLE, Vir–NBI, and NBI modes. (A1), (A2) and (A3) are WLE images, (B1), (B2) and (B3) are Vir–NBI images, and (C1), (C2) and (C3) are real NBI images.

Two endoscopy experts evaluated the image quality of WLE, NBI, and Vir–NBI based on consultation (Table 2; Figure 4). In terms of surface structure evaluation, 8 (7.2%) WLE images, 44 (39.6%) NBI images, and 51 (45.9%) Vir–NBI images were rated as good. Compared with WLE images, both NBI images (Steel–Dwaas test, *p* < 0.001) and Vir–NBI images (Steel–Dwaas test, *p* < 0.001) showed better observation results. In terms of evaluating blood vessels, 11 (9.9%) WLE images, 39 (35.1%) NBI images, and 31 (27.9%) Vir–NBI images were rated as good. Compared with WLE images, both NBI images (Steel–Dwaas test, *p* < 0.001) and Vir–NBI images (Steel–Dwaas test, *p* < 0.001) showed better observation results. In terms of evaluating lesion margins, 11 (9.9%) WLE images, 39 (35.1%) NBI images, and 33 (29.7%) Vir–NBI images were rated as good. Compared with WLE images, both NBI images (Steel–Dwaas test, *p* < 0.001) and Vir–NBI images (Steel–Dwaas test, *p* < 0.001) showed better results.

**Table 2.** Comparison of endoscopic experts’ evaluation of WLE, NBI, and Vir–NBI images quality.

	Quality evaluation			Statistical analysis	
Image mode	Poor	Moderate	Good	Friedman test *P* value	Steel–Dwass test *P* value
**Surface structure, *n***					
WLE	21	82	8		<.001^a^
NBI	6	61	44	<.001	0.608^b^
Vir–NBI	2	58	51		<.001^c^
**Surface blood vessels, *n***					
WLE	52	48	11		<.001^a^
NBI	17	55	39	<.001	0.759^b^
Vir–NBI	15	65	31		<.001^c^
**Lesion margins, *n***					
WLE	32	68	11		<.001^a^
NBI	8	64	39	<.001	0.735^b^
Vir–NBI	8	70	33		<.001^c^

WLE, white light endoscopy. NBI, narrow band imaging. Vir–NBI, virtual–narrow band imaging.

^a^WLE vs NBI.

^b^NBI vs Vir–NBI.

^c^Vir–NBI vs WLE.

### 3.3. Comparison of diagnostic ability of endoscopists observing different modes of endoscopic images for EGC

**Figure 5.**
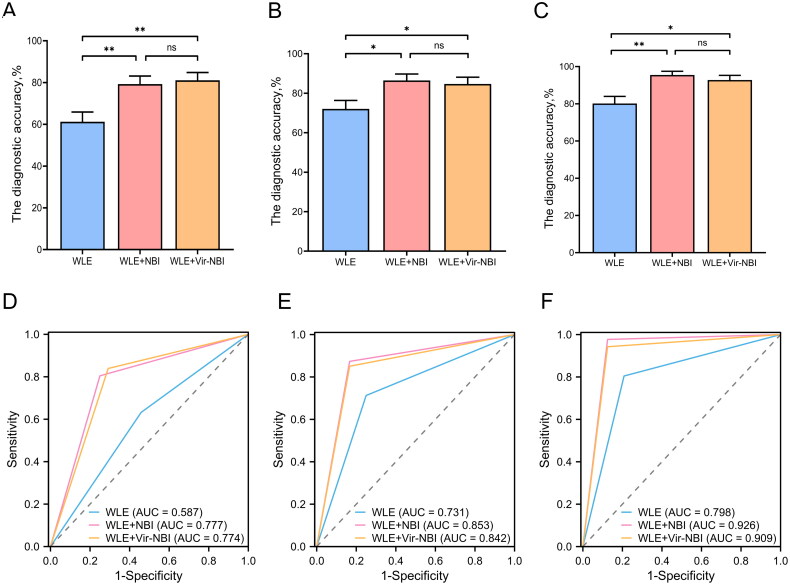
Different levels of endoscopists use different modes of endoscopic images to judge EGC. (A) The diagnostic accuracy of junior endoscopists. (B) The diagnostic accuracy of intermediate endoscopists. (C) The diagnostic accuracy of senior endoscopists. (D) ROC curve of diagnosis by junior endoscopists. (E) ROC curve of diagnosis by intermediate endoscopists. (F) ROC curve of diagnosis by senior endoscopists.

The diagnostic results of endoscopists with varying years of experience can be seen in Table 3 and Figure 5. For junior endoscopists, the accuracy of diagnosing EGC by observing WLE images alone was 61.26%, while the accuracy of diagnosing EGC with WLE + NBI images was 79.28%, and the accuracy of diagnosing EGC with WLE + Vir–NBI images was 81.08%. The diagnostic accuracy of simultaneous observation of WLE + NBI and WLE + Vir – NBI images was significantly higher than that of observing WLE images alone (*p* < 0.001, *p* < 0.001), while there was no significant difference between the simultaneous observation of WLE + NBI images and WLE + Vir – NBI images. For intermediate endoscopists, the accuracy of diagnosing EGC by observing WLE images alone was 72.07%, while the accuracy of diagnosing EGC with WLE + NBI images was 86.49%, and the accuracy with WLE + Vir – NBI images was 84.68%. Similar to the junior endoscopists, the diagnostic accuracy of simultaneous observation of WLE + NBI images and WLE + Vir–NBI images was significantly higher than that of observing WLE images alone (*p* < 0.05, *p* < 0.05), with no significant difference between simultaneous observation of WLE+NBI images and WLE + Vir–NBI images. For senior endoscopists, the accuracy of diagnosing EGC by observing WLE images alone was 80.18%, while the accuracy of diagnosing EGC using WLE+NBI images is 95.50%, and the accuracy with WLE + Vir–NBI images is 92.79%. The diagnostic accuracy of simultaneous observation of WLE+NBI images and WLE + Vir – NBI images was significantly higher than that of observing WLE images alone (*p* < 0.001, *p* < 0.05). There was no significant difference in the accuracy of diagnosing EGC between observing WLE images alone and observing WLE + Vir–NBI images simultaneously.

**Table 3.** Comparison of the diagnostic ability of endoscopists with different years of experience in EGC by observing different modes of endoscopic images.

	Accuracy % (95% CI)	Sensitivity % (95% CI)	Specificity % (95% CI)	AUC (95%CI)
**Junior**				
WLE	61.26 (51.97,69.80)	63.22 (52.73,72.59)	54.17 (35.07,72.11)	0.59 (0.47,0.70)
WLE +NBI	79.28 (70.82,85.78)	80.46 (70.92,87.43)	75.00 (55.10,88.00)	0.78 (0.68,0.87)
WLE +Vir–NBI	81.08 (72.80,87.28)	83.91 (74.78,90.17)	70.83 (50.83,85.09)	0.77 (0.67,0.87)
**Intermediate**				
WLE	72.07 (63.10,79.57)	71.26 (61.02,79.71)	75.00 (55.10,88.00)	0.73 (0.63,0.83)
WLE + NBI	86.49 (78.90,91.64)	87.36 (78.76,92.79)	83.33 (64.15,93.32)	0.85 (0.76,0.93)
WLE + Vir–NBI	84.68 (76.84,90.21)	85.06 (76.10,91.05)	83.33 (64.15,93.32)	0.84 (0.75,0.92)
**Senior**				
WLE	80.18 (71.81,86.53)	80.46 (70.92,87.43)	79.17 (59.53,90.76)	0.80 (0.70,0.89)
WLE + NBI	95.50 (89.89,98.06)	97.70 (92.00,99.37)	87.50 (69.00,95.66)	0.93 (0.85,0.99)
WLE + Vir–NBI	92.79 (86.42,96.30)	94.25 (87.24,97.52)	87.50 (69.00,95.66)	0.91 (0.83,0.97)

WLE white light endoscopy, NBI narrow band imaging, Vir–NBI virtual–narrow band imaging, AUC area under curve; 95% CI 95% confidence interval.

### 3.4. Comparison of the area concordance rate between WLE and Vir–NBI images

**Figure 6.**
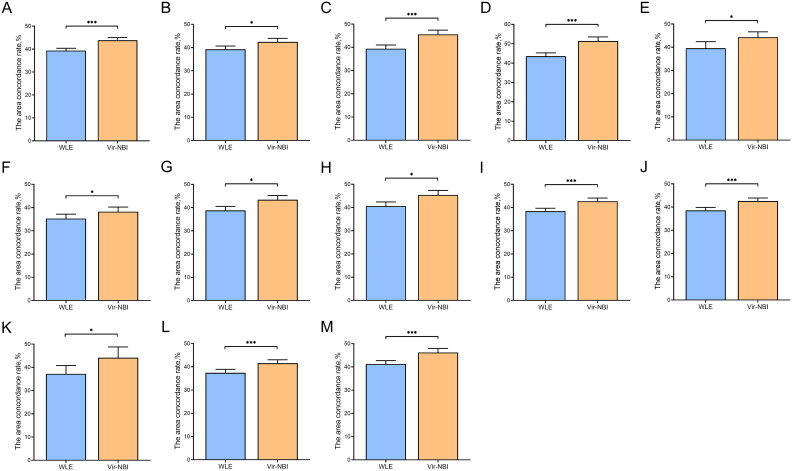
Comparison of the area concordance rate between WLE and Vir–NBI images. (A) All. (B) Specimen size ≤3.5 cm. (C) Specimen size >3.5 cm. (D) Elevated type. (E) Flat type. (F) Depressed type. (G) Middle 1/3. (H) Lower 1/3. (I) Differentiated type. (J) Intraepithelial neoplasia + intramucosal carcinoma. (K) Submucosal carcinoma. (L) Junior endoscopists. (M) Senior endoscopists (*p < 0.05, **p < 0.01, ***p < 0.001).

Overall, Vir–NBI images showed a significantly higher area concordance rate than WLE images (43.85% vs 39.32%, *p* < 0.001) (Table 4; Figure 6). In the subgroup analysis, the area concordance rate of Vir–NBI images with specimen size ≤3.5 cm and >3.5 cm was significantly higher than that of WLE images (42.46% vs. 39.25%, *p* = 0.013) (45.57% vs 39.40%, *p* < 0.001). The area concordance rate of elevated, flat and depressed type lesions in Vir–NBI images was significantly higher than that in WLE images (51.37% vs 43.58%, *p* < 0.001) (44.31% vs 39.57%, *p* = 0.030) (38.22% vs 35.25%, *p* = 0.035). The area concordance rate of lesions located in the middle 1/3 and lower 1/3 of Vir–NBI images was significantly higher than that in WLE images (43.38% vs 38.82%, *p* = 0.002) (45.42% vs 40.63%, *p* = 0.002). The area concordance rate of differentiated EGC in Vir–NBI images was significantly higher than that in WLE images (42.68% vs 38.41%, *p* < 0.001). The area concordance rate of epithelial neoplasia or mucosal layer and submucosal layer EGC in Vir–NBI images was significantly higher than that in WLE images (42.58% vs 38.57%, *p* < 0.001) (44.15% vs 37.19%, *p* = 0.041). The area concordance rate between junior and senior endoscopists in Vir–NBI images was significantly higher than that in WLE images (41.51% vs 37.39%, *p* < 0.001) (46.19% vs 41.24%, *p* < 0.001).

**Table 4.** Comparison of the area concordance rate between WLE and Vir–NBI images.

	WLE% (95%CI)	Vir–NBI% (95% CI)	*P*
**All**	39.32 (38.27,40.37)	43.85 (42.69 45.02)	<0.001
**Specimen size, *n***			
≤3.5 cm (*n* = 59)	39.25 (37.85,40.62)	42.46 (40.95 43.96)	0.013
>3.5 cm (*n* = 48)	39.40 (37.81,41.00)	45.57 (43.76,47.39)	<0.001
**Morphology^a^, *n***			
Elevated type (*n* = 31)			
Flat type (*n* = 16)	43.58 (41.88,45.27)	51.37 (49.23,53.50)	<0.001
Depressed type (*n* = 36)	39.57 (36.75,42.39)	44.31 (42.01,46.61)	0.030
Mixed type (*n* = 24)	35.25 (33.34,37.17)	38.22 (36.23,40.21)	0.035
Mixed type (*n* = 12)	39.75 (37.67,41.83)	42.30 (40.00,44.58)	0.206
**Location, *n***			
Upper 1/3 (*n* = 25)	38.26 (36.20,40.33)	42.39 (39.93,44.84)	0.075
Middle 1/3 (*n* = 45)	38.82 (37.13,40.51)	43.38 (41.55,45.26)	0.002
Lower 1/3 (*n* = 37)	40.63 (38.86,42.39)	45.42 (43.48,47.35)	0.002
**Histologic type of EGC^b^, *n***			
Differentiated type (*n* = 73)	38.41 (37.13,39.68)	42.68 (41.30,44.07)	<0.001
Undifferentiated type (*n* = 10)	38.40 (34.95,41.86)	43.44 (39.85,47.04)	0.085
**Invasion depth of EGC, *n***			
Intraepithelial neoplasia^a^			
Intramucosal carcinoma (*n* = 73)	38.57 (37.30, 39.83)	42.58 (41.26,43.92)	<0.001
Submucosal carcinoma (*n* = 10)	37.19 (33.61,40.76)	44.15 (39.55,48.75)	0.041
**Endoscopists, *n***			
Junior endoscopists (*n* = 3)	37.39 (35.90, 38.89)	41.51 (40.03, 43.01)	<0.001
Senior endoscopists (*n* = 3)	41.24 (39.79, 42.69)	46.19 (44.42, 47.96)	<0.001

EGC early gastric cancer, WLE white light endoscopy, Vir–NBI virtual–narrow band imaging, 95% CI 95% confidence interval.

^a^According to the Paris endoscopic classification.

^b^According to the Nakamura classification.

### 3.5. Comparison of successful whole lesion diagnosis between WLE and Vir–NBI images

**Figure 7.**
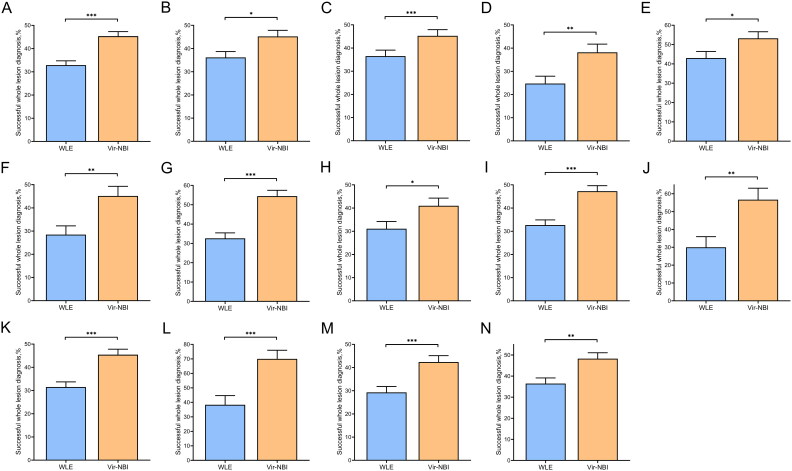
Comparison of successful whole lesion diagnosis between WLE and Vir–NBI images. (A) All. (B) Specimen size ≤3.5 cm. (C) Specimen size >3.5 cm. (D) Elevated type. (E) Depressed type. (F) Mixed type. (G) Middle 1/3. (H) Lower 1/3. (I) Differentiated type. (J) Undifferentiated type. (K) Intraepithelial. Neoplasia + intramucosal carcinoma. (L) Submucosal carcinoma. (N) Junior endoscopists. (N) Senior endoscopists (*p < 0.05, **p < 0.01, ***p < 0.001).

Overall, Vir–NBI images showed a significantly higher successful whole lesion diagnosis than WLE images (45.33% vs 32.87%, *p* < 0.001) (Table 5; Figure 7). In subgroup analysis, successful whole lesion diagnosis with specimen size≤ 3.5 cm and >3.5 cm in Vir–NBI images was significantly higher than that in WLE images (45.40% vs 36.49%, *p* = 0.011) (45.24% vs 28.57%, *p* < 0.001). Successful whole lesion diagnosis of elevated, depressed and mixed type lesions in Vir–NBI images was significantly higher than that in WLE images (38.17% vs 24.73%, *p* = 0.004) (53.24% vs 43.06%, *p* = 0.027) (45.14% vs 28.47%, *p* = 0.003). Successful whole lesion diagnosis of lesions located in the middle 1/3 and lower 1/3 of Vir–NBI images was significantly higher than that of WLE images (54.44% vs 32.59%, *p* < 0.001) (40.99% vs 31.08%, *p* = 0.030). Successful whole lesion diagnosis of differentiated and undifferentiated EGC in Vir–NBI images was significantly higher than that in WLE images (47.26% vs 32.65%, *p* < 0.001) (56.67% vs 30.00%, *p* = 0.003). Successful whole lesion diagnosis of epithelial neoplasia or mucosal layer and submucosal layer EGC in Vir–NBI images was significantly higher than that in WLE images (45.43% vs 31.51%, *p* < 0.001) (70.00% vs 38.33%, *p* < 0.001). Successful whole lesion diagnosis between junior and senior endoscopists in Vir–NBI images was significantly higher than that in WLE images (42.37% vs 29.28%, *p* < 0.001) (48.29% vs 36.45%, *p* = 0.004).

**Table 5.** Comparison of successful whole lesion diagnosis between WLE and Vir–NBI images.

	WLE% (95% CI)	Vir–**NBI% (95% CI)**	*P*
**All**	32.87 (29.22,36.51)	45.33 (41.47,49.19)	<0.001
**Specimen size, *n***			
≤3.5cm (*n* = 59)	36.49 (31.41,41.58)	45.40 (40.15,50.66)	0.011
>3.5cm (*n* = 48)	28.57 (23.38,33.77)	45.24 (39.52,50.96)	<0.001
**Morphology^b^, *n***			
Elevated type (*n* = 31)	24.73 (18.47,30.99)	38.17 (31.13,45.22)	0.004
Flat type (*n* = 16)	32.29 (22.77,41.82)	41.67 (31.62,51.57)	0.178
Depressed type (*n* = 36)	43.06 (36.40,49.71)	53.24 (46.53,59.95)	0.027
Mixed type (*n* = 24)	28.47 (21.01,35.93)	45.14 (36.91,53.36)	0.003
**Location, *n***			
Upper 1/3 (*n* = 25)	36.00 (28.23,43.77)	35.33 (27.60,43.07)	0.904
Middle 1/3 (*n* = 45)	32.59 (26.97,38.22)	54.44 (48.44,60.42)	<0.001
Lower 1/3 (*n* = 37)	31.08 (24.95,37.22)	40.99 (34.47,47.51)	0.030
**Histologic type of EGC^b^, *n***			
Differentiated type (*n* = 73)	32.65 (28.24,40.69)	47.26 (42.57,51.95)	<0.001
Undifferentiated type (*n* = 10)	30.00 (18.06,41.94)	56.67 (43.76,69.58)	0.003
**Invasion depth of EGC (%)**			
Intraepithelial neoplasia^a^			
Intramucosal carcinoma (*n* = 73)	31.51 (27.14,35.87)	45.43 (40.75,50.12)	<0.001
Submucosal carcinoma (*n* = 10)	38.33 (25.67,51.00)	70.00 (58.06,81.94)	<0.001
**Endoscopists**			
Junior endoscopists (*n* = 3)	29.28 (24.28,34.29)	42.37 (36.93,47.80)	<0.001
Senior endoscopists (*n* = 3)	36.45 (31.16,41.74)	48.29 (42.79,53.78)	0.004

EGC early gastric cancer, WLE white light endoscopy, Vir–NBI virtual–narrow band imaging, 95% CI 95% confidence interval.

^a^According to the Paris endoscopic classification.

^b^According to the Nakamura classification.

## 4. Discussion

To our knowledge, this is the first study to generate Vir–NBI images based on stable diffusion for the diagnosis of EGC. The results demonstrate that Vir–NBI images significantly outperform WLE images in identifying surface structures and lesion edges, closely resembling real NBI images. Furthermore, compared to observing WLE images alone, junior and intermediate endoscopists showed a significant improvement in EGC diagnostic accuracy when simultaneously observing WLE + Vir–NBI images. Finally, Vir–NBI images not only offer superior delineation of lesion boundaries compared to WLE images but also assist endoscopists in identifying the entire lesion more clearly.

In recent years, stable diffusion has made significant strides in multiple fields of medical image processing. It has demonstrated notable progresses in tasks such as image translation, image reconstruction, image classification, and segmentation, showcasing strong potential in these areas [23]. Lyu et al. [25] applied stable to solve the translation problem from MRI to CT, and the results showed that stable diffusion outperform CycleGAN based methods in terms of image quality and structural similarity. Another study proposed a medical image classification method based on stable diffusion, which proved effective in assessing the grading of placental maturity, skin lesions and diabetes retinopathy [26]. These studies indicate that stable diffusion not only effectively handles complex features in medical images but also offers unique advantages in tasks such as cross modal data, unsupervised learning, and image generation. Its flexibility and powerful generation capability make stable diffusion widely applicable in medical image analysis, especially in situations with insufficient data or scarce labels. It can effectively improve image quality, enhance data representation, and provide strong support for precision medicine.

Although stable diffusion has demonstrated advantages in image generation and transformation tasks, it still has some technical limitations. For example, the large parameter count of stable diffusion requires a substantial memory to store weights and intermediate results. This high demand may limit the application of stable diffusion on devices with restricted resources. Despite advancements in hardware, the computational and storage overhead of stable diffusion remains a challenge for some embedded devices or low budget computing environments. In addition, due to its own characteristics, the speed of image conversion of stable diffusion is limited. In this study, the average time of converting WLE images into Vir–NBI images was 27.8s, and endoscopists could not get the converted images immediately. However, considering that the conventional endoscopy time often reached more than 5 min, stable diffusion could still ensure that endoscopists could complete the observation and diagnosis of Vir–NBI images during the examination.

In this study, we generated Vir–NBI images from WLE images based on stable diffusion, and the generated Vir–NBI images closely resemble real NBI images to the naked eye. According to the evaluation and assessment of endoscopists, Vir–NBI images provided better lesions visibility and more accurate identification of EGC compared to WLE images.

The area concordance rate of WLE and Vir–NBI images in the lesion area reached 39.32% and 43.85%, respectively. Based on actual clinical experience, both values seem slightly lower, which may be due to the relatively limited lesion information presented in static images, leading to insufficient ability of endoscopists in evaluation. A study from Japan [14]showed that the area concordance rate of WLE and NBI images in EGC were 37.2% and 43.1%, respectively. Additionally, a study by Suzhuki et al. [21]showed that the area concordance rate of WLE and VIC images in gastric tumors were 48.5% and 44.1%, respectively. The differences between this study and the above research results are small, suggesting that using WLE images as a control for Vir–NBI images in this study is reasonable.

This study has several advantages. First, through Stable Diffusion, WLE images can be easily and directly converted into Vir–NBI images that closely resemble real NBI images, enabling more endoscopists in medical institutions to observe lesions clearly on high-quality images. Second, Vir–NBI images effectively support the accurate diagnosis of EGC, particularly for less experienced endoscopists. Third, Vir–NBI images assist endoscopists in fully identifying the entire lesion, thereby achieving complete resection.

However, this study also has some limitations. First, we used static image pairs to verify the effect, but in clinical practice, endoscopists typically observe lesions in dynamic examination videos. Our goal is to collect more endoscopic video data in the future to further evaluate the effectiveness of stable diffusion conversion. Second, all endoscopic images collected in this study were obtained from Olympus 260 or 290 series gastroscopy systems, with no endoscopic images from other series brands such as Fuji. In the future, we aim to expand our dataset to include images from other manufacturers and develop an AI-powered virtual electronic staining endoscopic system that is not limited by suppliers. Third, although this study shows that the Vir–NBI images generated by stable diffusion perform well in diagnosing EGC, it is important to note that Vir–NBI images are not real NBI images. As a new imaging modality, endoscopists will need a certain learning period to become proficient in its application.

In summary, the Vir–NBI images generated by stable diffusion are helpful for the observation and diagnosis of EGC. In the future, we will continue to optimizing the model structure, reducing video memory usage, and enhancing computational efficiency, with the goal of enabling Vir–NBI image conversion in dynamic inspection videos.

## Supplementary Material

Supplemental Material

## Data Availability

The datasets used and/or analysed during the current study available from the corresponding author on reasonable request.
