# Supplementary material for: Virtual NBI image synthesis using stable diffusion for enhanced recognition of early gastric cancer: a technical validation study
Source: Ann Med. 2025 Jun 28;57(1):2523565. doi: 10.1080/07853890.2025.2523565 (PMC12207768; doi:10.1080/07853890.2025.2523565)
Supplement: Supplemental Material [file IANN_A_2523565_SM5330.docx]

**Acronyms and abbreviations**

NBI: narrow band imaging

EGC: early gastric cancer

WLE: white light endoscopy

Vir-NBI: virtual narrow band imaging

ESD: endoscopic submucosal dissection

GC: gastric cancer

AI: artificial intelligence

VIC:Virtual indigo carmine

LoRA: low-Rank Adaptation

FID: Fréchet Inception Distance

LPIPS: Learned Perceptual Image Patch Similarity

ArtFID: Artifact-aware FID

SSIM: Structural Similarity Index Measure
